# Bioresources inner-recycling between bioflocculation of *Microcystis aeruginosa* and its reutilization as a substrate for bioflocculant production

**DOI:** 10.1038/srep43784

**Published:** 2017-03-02

**Authors:** Liang Xu, Mingxin Huo, Caiyun Sun, Xiaochun Cui, Dandan Zhou, John C. Crittenden, Wu Yang

**Affiliations:** 1 School of Environment, Northeast Normal University, Changchun 130117, China; 2 Jilin Institute of Chemical Technology, Jilin, 132022, China; 3 Brook Byers Institute for Sustainable Systems, and School of Civil & Environmental Engineering, Georgia Institute of Technology, Atlanta, GA 30332, USA

## Abstract

Bioflocculation, being environmental-friendly and highly efficient, is considered to be a promising method to harvest microalgae. However, one limitation of this technology is high expense on substrates for bioflocculant bacteria cultivation. In this regard, we developed an innovative method for the inner-recycling of biomass that could harvest the typical microalgae, *Microcystis aeruginosa*, using a bioflocculant produced by *Citrobacter* sp*. AzoR-1*. In turn, the flocculated algal biomass could be reutilized as a substrate for *Citrobacter* sp*. AzoR-1* cultivation and bioflocculant production. The experimental results showed that 3.4 ± 0.1 g of bioflocculant (hereafter called MBF-12) was produced by 10 g/L of wet biomass of *M. aeruginosa* (high-pressure steam sterilized) with an additional 10 g/L of glucose as an extra carbon source. The efficiency of MBF-12 for *M. aeruginosa* harvesting could reach ~95% under the optimized condition. Further analysis showed that MBF-12, dominated by ~270 kDa biopolymers, contributed the bioflocculation mechanisms of interparticle bridging and biosorption process. Bioflocculant synthesis by *Citrobacter* sp*. AzoR-1* using microalga as a substrate, including the polyketide sugar unit, lipopolysaccharide, peptidoglycan and terpenoid backbone pathways. Our research provides the first evidence that harvested algae can be reutilized as a substrate to grow a bioflocculant using *Citrobacter* sp*. AzoR-1*.

In recent years, microalgal blooms have drawn substantial attention, particularly because of the threat they pose to human health and the environment^1^. *Microcystis aeruginosa (M. aeruginosa*) is a ubiquitous toxin-producing cyanobacteria present in the aquatic environment^2^. *M. aeruginosa* can reduce dissolved oxygen levels, cause discoloration of receiving water (red tide) and produce odors and toxins that pose hazards to human health and aquatic ecosystems^3^. Indeed, from another perspective, microalgae are promising and new biomass resources for lots of high-value applications, i.e. triacylglycerol, bioalcohols (e.g., ethanol and butanol), polyunsaturated fatty acids (e.g., eicosapentaenoic acid, and docosahexaenoic acid), and pigments (e.g., lutein and chlorophyll)^4^. Combining and considering above aspects, one of the promising ways to achieve microalgal biomass would be collecting the microalgal cells that bloomed and suspended in the aquatic environment^5^. To address this, flocculation technologies have to be applied for either waterbody remediation or microalgae biomass recovery^6^.

However, the high economic cost for separating microalgae biomass from water is still a bottleneck. Traditional bioflocculants, such as ferric salts or aluminum salts, have been studied to flocculate the microalgal cells^6^,^7^. Although the removal efficiency for traditional flocculants was quite high in many studies, these bioflocculants have certain disadvantages for long-term use. Residual aluminum in treated water, either in the supernatant or in sludge, is difficult to remove and sometimes exceeds the upper limit of water standards, causing a threat to human health^8^. In this context, a great deal of research has been devoted to the use of natural materials as flocculants, such as clays^9^, chitosan^10^, and cationic starch^11^,^12^, which can be biodegraded, and thus are safer for humans and the ecosystem. Especially, flocculants produced by microalgae, bacteria or fungus are more attractive for microalgae recovery, due to its safety, biodegradability and non-secondary pollution^13^,^14^. However, the high cost of bioflocculants takes the cost prohibitive issue, in which the high cost on the substrates (e.g., carbon sources and nitrogen sources) that are used for the cultivation of bioflocculants producing microorganism is the major challenge. Previous studies used crop stalks and kitchen wastes to produce bioflocculants, but the bioflocculant yield was unsatisfactory^15^,^16^, and the production conditions were critical^17^,^18^. Straw contains a large amount of cellulose, which can be used as a carbon source. However, during bioflocculant production, cellulose-degrading bacteria need to be added, which increases the competition between the microbial populations and causes an antagonistic effect^16^.

The microalgae, *M. aeruginosa*, we intend to flocculate are potentially a promising substrate for bioflocculant bacteria cultivation. The carbon and nitrogen levels in the *M. aeruginosa* biomass reached 22 to 28% and 4 to 5%, respectively^19^, indicating that the biomass can provide a potential nutrition source for bioflocculant production. Thus, we hypothesized a clue for the inner-recycling of biomass that could harvest the typical microalgae, *M. aeruginosa*, using a bioflocculant produced by bacterium (e.g., *Citrobacter* sp*. AzoR-1* in this work). In turn, the algal biomass was utilized as a substrate for *Citrobacter* sp*. AzoR-1* cultivation and bioflocculant production.

To confirm above hypothesis, we cultivated a bioflocculant-producing bacterium, *Citrobacter* sp*. AzoR-1,* which produces a bioflocculant with a high harvest efficiency for a typical microalga, *M. aeruginosa.* Next, we examined the flocculated *M. aeruginosa* for bioflocculant production and determined the optimal bioflocculant conditions and the mechanisms of flocculation using the bioflocculant. The metabolic and gene expression profiles of the microorganisms during the microalgal consumption were identified through transcription analyses, to reveal the pathways underlying the bioflocculant production. What is novel would be the bioresources inner-recycling between bioflocculation of *Microcystis aeruginosa* and its reutilization as a substrate for bioflocculant production. This work provided a promising and new clue for efficiently and economically treating microalgae blooms.

## Results

### Harvesting of M. aeruginosa by the produced bioflocculant

RSM provided response surfaces and contour plots to study the interactions between the operational parameters and removal efficiency. All of the selected optimum solutions retained the desired simulations of the removal efficiency (see Table S4). Predicted data achieved from the response surface methodology (RSM) under the optimal conditions were compared with the experimental proving results to validate the model. The experimental verification values observed for the removal efficiency ranged from 91.68% to 97.21% (see Table S4). Deviations between the experimental and the predicted values are all within 2%, indicating that the model fitted the experimental data well (see Table S4). Furthermore, to intestate the liner variable’s impact on the flocculation efficiency with other variables fixed in median, the experimental verification values were also fixed the prediction curve as shown in Figure S1. The optimal *M. aeruginosa* flocculation conditions by MBF-12 were from 10 to 30 °C in temperature, 12.7 mg/L in dosage, 1.2 hours in settling time and pH <8 (see Figure S1).

**Figure 1:**
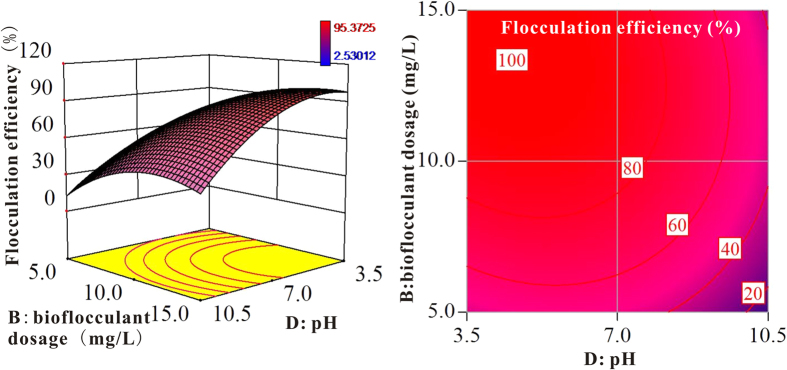
Surface responses showing the interactive effects of selected variables on the flocculation efficiency.

The highest removal efficiency of *M. aeruginosa* was 95.37%, and the lowest was 2.53%. Two linear terms, the pH and the MBF-12 dose, had significant effects on the flocculation efficiency (Fig. 1). The optimum MBF-12 dosage was 12.70 mg/L for 10^9^ algal cells per liter. The removal efficiency began to slightly decrease at higher bioflocculant doses because the formation of negatively charged flocculants led to charge protection^19^. Furthermore, for a fixed MBF-12 dose of 10 mg/L, the removal efficiency decreased from 87.09% to 7.21% when the pH was increased from 8 to 13. Such significant effect of pH are related to the functional groups changing on bioflocculant biopolymers under alkaline conditions^20^,^21^,^22^,^23^,^24^, which will be discussed later.

### Reutilization of flocculated M. aeruginosa as a substrate for bioflocculant production

**Figure 2:**
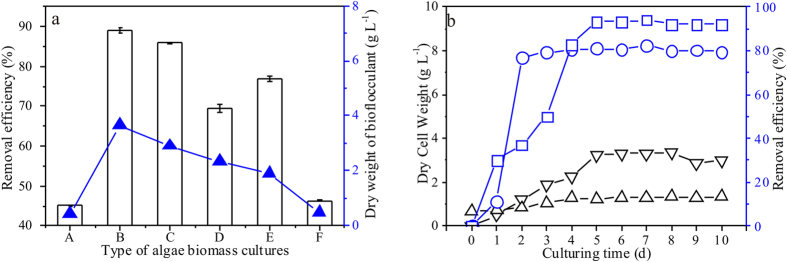
(**a**) Bioflocculant productivity with algal biomasses cultivated under various culture conditions (Culture A-F), and the *M. aeruginosa* removal by the produced bioflocculant; (**b**) Growth of *Citrobacter Aroz-1* in PT-1 and the algal biomass recovered from Culture-C at various points during the cultivation and the corresponding removal efficiencies of *M. aeruginosa* cells. 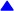 Dry weight of the bioflocculant (g L^−1^); bars represent the corresponding removal efficiencies of *M. aeruginosa* in flocculant dosage of 0.1 g; ▽ Dry *Citrobacter Aroz-1* cell weight in PT-1 culture; Δ Dry *Citrobacter Aroz-1* cell weight in *M. aeruginosa* biomass culture. 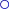 Flocculation efficiency of *M. aeruginosa* by the bioflocculant obtained from PT-1 culture; 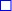 Flocculation efficiency of *M. aeruginosa* by the bioflocculant obtained from the algal biomass recovered from Culture-C.

Flocculated *M. aeruginosa* was reused as the main substrate for bioflocculant production in Culture A-F to select an optimal media. Then, Culture-C was selected for all the subsequent bioflocculant (MBF-12) production protocols, as it achieved both a high removal efficiency and low glucose consumption (see Fig. 2a). As shown in Fig. 2(a), Culture-B and Culture-C achieved the highest bioflocculant production efficiencies, which reached 3.85 and 2.9 g L^−1^ (dry weight per Liter culture), respectively. In contrast, the production efficiencies was the lowest and no more than 1 g L^−1^ for both of Culture-A and Culture-F. Bioflocculant productivity in PT-1 and Culture-C at different growth stage were also studied, as is shown in Figure S3, the highest productivity started at 4th day, which suggest that, in both PT-1 and Culture-C, the synthesis of bioflocculant is mainly in the late stage of microbial growth. The maximum yeild of bioflocculant was 4.3 g L^−1^ in PT^−1^ and 3.8 g L^−1^(dry weight per Liter culture) in Culture-C, respectively.

The biomass production (presented as the dry cell weight) and removal efficiencies obtained from Culture-C and PT-1 cultivation were compared. PT-1 was used as a background that was cultivated without using algae. Interestingly, even though the cell proliferation rate in Culture-C was 50% lower than that in PT-1, the removal efficiency of the bioflocculant produced in Culture-C was 15% higher than that of PT-1 (see Fig. 2b), at MBF-12 dosage of 12 mg/L; bioflocculation time of 0.1 hour and settling time of 1.2 hours. The two cultures apparently produced bioflocculants with different characteristics. *M. aeruginosa* possesses a high protein content and a low sugar content (18% w/w)^25^, which signifies that the extra glucose included in Culture-C was the main reason for the more efficient bioflocculant production in Culture-C.

### Characteristics of the bioflocculant (MBF-12)

**Figure 3:**
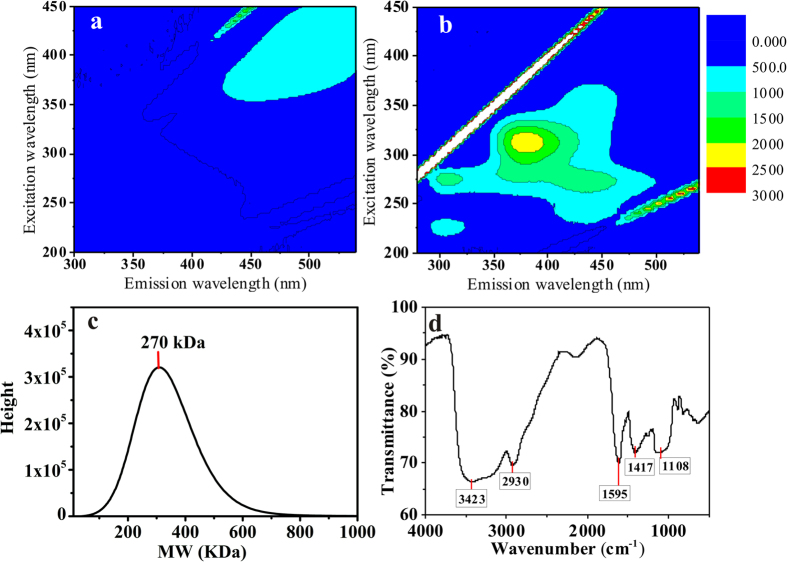
MBF-12 bioflocculant characterization (**a**) Fluorescence excitation emission matrix contours of the MBF-12 produced by *Citrobacter Aroz-1* with PT-1culture. (**b**) Fluorescence excitation emission matrix contours of the MBF-12 produced by *Citrobacter Aroz-1* with Culture-C. (**c**) The molecular weight (MW) of MBF-12 form Culture-C. (**d**) Fourier-transform infrared spectra (FTIR) of the purified MBF-12 from Culture-C.

The microbial bioflocculant of *Citrobacter* sp*. AzoR-1,* termed MBF-12, was characterized by EEM, Molecular weight and FTIR spectrum. The EEM spectrum (Fig. 3a) shows that MBF-12 from PT-1 culture mainly contained polycarboxylate compounds^22^,^23^ (Ex/Em = 300–450/400–550 nm)^21^,^22^. The intensity of the fluorophores of the polycarboxylate-type compound was the highest, and no polysaccharide (Ex/Em = 300–450/350–400 nm) or polyaromatic acid compounds (Ex/Em = 250–300/400–550 nm) were found. In contrast, MBF-12 from Culture-C had a different composition (Fig. 3b), in which contained more protein-like compounds and polysaccharide-like organics. To be specified, these compounds include polysaccharide (Ex/Em = 300–450/350–400 nm), polyaromatic-type polysaccharides (Ex/Em = 250–300/400–550 nm), and tyrosine/tryptophan amino acids (Ex/Em = 200–250/300–400 nm).

MBF-12 is a long-chain biopolymer which molecular weight was 270 kDa (see Fig. 3c), so it is long enough to perform bridging between *M. aeruginosa* cells. Notably, MBF-12 was unfolded in solution and with a size larger than 10 μm according to its SEM image (see Figure S2), which should contribute to a more effective flocculation by bridging the *M. aeruginosa* cells.

The Fourier-transform infrared (FTIR) spectrum of purified MBF-12, which was generated from Culture-C, exhibited a broad hydroxyl stretching peak at 3423 cm^−1^ and an amine band at 1630–1550 cm^−1^, as shown in Fig. 3d. The adsorption peak at 2930 cm^−1^ indicated the C-H stretching vibration. Strong absorption peaks present in the range of 1100–1200 cm^−1^ are typical peaks for proteoglycans^25^.

### Bioflocculant metabolic pathways

Metabolism-related genes comprised a significant portion of the annotated non-redundant sequences, as found in other microbial transcriptomes^26^,^27^,^28^. More than 50% of the 4,961 non-redundant sequences were predicted to be associated with metabolism, and many of the metabolic genes were predicted to be associated with polysaccharide biosynthesis, reflecting the bioflocculant products of the strain.

**Figure 4:**
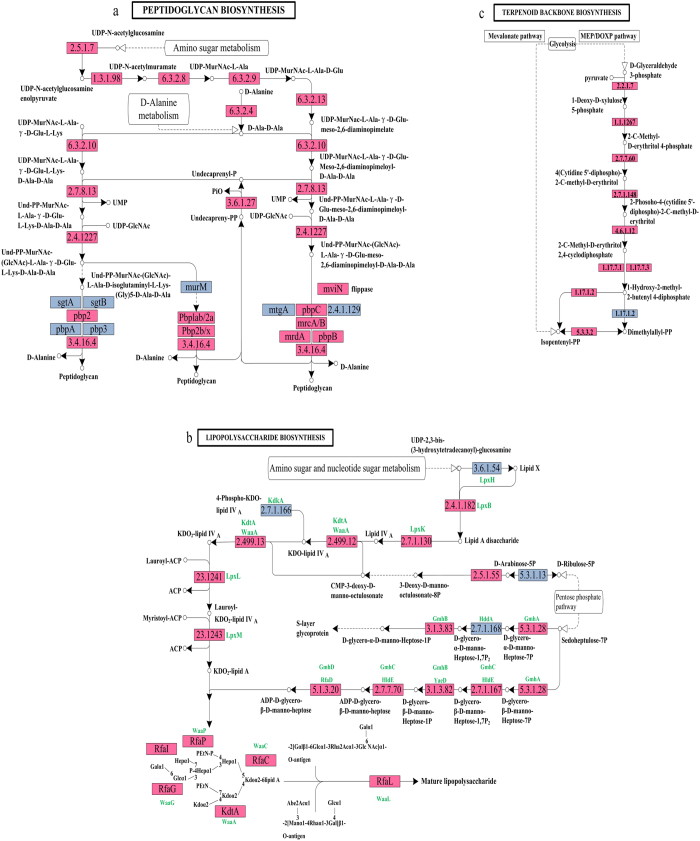
The peptidoglycan and terpenoid backbone biosynthesis pathways used by *Citrobacter* sp*. Aroz-1* with Culture-C (upregulated genes are marked in red, and non-expressed genes are marked in blue).

A search for enzymes that are potentially associated with the biosynthesis of flocculation-related polysaccharides retrieved 57 non-redundant sequences (the EC numbers are shown in Supplementary Information Table S5). Because the genes for the final conversion of saturated bioflocculants to hydrocarbon end products remain unidentified, the transcriptome data included putative genes associated with unsaturated hydrocarbons. Such genes are related to bioflocculant biosynthesis, including the polyketide sugar unit, lipopolysaccharide, peptidoglycan, and terpenoid backbone (Table S5 and Fig. 4). Genes related to these bioflocculant biosynthesis pathways were classified into different functional categories using the Kyoto Encyclopedia of Genes and Genomes (KEGG) Automatic Annotation Server (KAAS) program, as shown in Fig. 4 and Table S5. The KEGG pathway database is copyrighted by Kanehisa laboratories^29^,^30^,^31^.

Polyketide sugar unit biosynthesis is catalyzed by dTDP-4-dehydrorhamnose reductase ([1.1.1.133]), as shown in Supplementary Table S5. Peptidoglycan biosynthesis was initiated from UDP-N-acetylglucosamine from amino sugar metabolism, and 29 genes participated in the complete pathway, as shown in Fig. 4a and Supplementary Table S5. The synthesis of UDP-MurNAc-L-Ala-D-Glu from UDP-MurNAc-L-Ala was catalyzed by UDP-N-acetylmuramoyl-L-alanyl-D-glutamate synthetase ([6.3.2.9]). Then, Und-PP-MurNAc-L-Ala-γ-D-Glu-meso-2,6-diaminopimeloyl-D-Ala-D-Ala synthesis was catalyzed by two key enzymes: UDP-N-acetylmuramoyl-tripeptide--D-alanyl-D-alanine ligase ([6.3.2.10]) and phospho-N-acetylmuramoyl-pentapeptide-transferase ([2.7.8.13]). Und-PP-MurNAc-L-Ala-γ-D-Glu-L-Lys-D-Ala-D-Ala and Undecapreny-PP are important precursors for peptidoglycan biosynthesis through different pathways. D-Alanine, derived from D-Alanine metabolism, is a mediator for the final synthesis of peptidoglycan by complex enzymes ([3.4.16.4]), such as D-alanyl-D-alanine carboxypeptidase, peptidase M15 and D-alanyl-D-alanine carboxypeptidase D-alanyl-D-alanine carboxypeptidase/endopeptidase.

Lipopolysaccharide biosynthesis can start from either sedoheptulose-7P via the pentose phosphate pathway or lipid X (as shown in Fig. 4b and Table S5), which is a common and necessary compound inside the cell. Sixteen of the identified genes participated in the lipopolysaccharide biosynthesis pathway, and an intermediate lipopolysaccharide was formed because the enzyme complex contained only lipopolysaccharide heptosyltransferase 1 ([2.4.-.-]), rfaP ([2.7.1.-]), transferase ([2.4.99.12]) and glucosyltransferase I RfaG ([2.4.1.-]). KDO2-lipid A and ADP-D-glycero-β-D-manno-heptose are two necessary precursors for lipopolysaccharide biosynthesis. The synthesis of both precursors was catalyzed by two distinct enzyme systems. KDO_2_-lipid A was synthesized from Lipid A disaccharide, which was derived from Lipid X, which involves five reactions and five enzymes: tetraacyldisaccharide 4′-kinase ([2.7.1.130]), transferases ([2.4.99.12] and [2.4.99.13]) and two hypothetical proteins ([2.4.1.-]). ADP-D-glycero-β-D-manno-heptose was synthesized from sedoheptulose-7P, which was obtained from the pentose phosphate pathway. The catalytic process also included five reactions and five enzymes: phosphoheptose isomerase ([5.3.1.28]), kinase/heptose 1-phosphate adenyltransferase ([2.7.1.167]), D,D-heptose 1,7-bisphosphate phosphatase ([3.1.3.82 3.1.3.83]), adenyltransferase ([2.7.7.70]) and ADP-L-glycero-D-mannoheptose-6-epimerase ([5.1.3.20]).

The MEP/DOXP pathway, rather than the mevalonate pathway, is the main pathway involved in terpenoid backbone biosynthesis (See Fig. 4c and Table S5,). Isopentenyl-PP was obtained from 1-hydroxy-2-methyl-2-butenyl 4-diphosphate through the MEP/DOXP pathway. In the MEP/DOXP pathway, 1-hydroxy-2-methyl-2-butenyl 4-diphosphate was formed by 4-hydroxy-3-methylbut-2-en-1-yl diphosphate synthase from 2-C-methyl-D-erythritol 2,4-cyclodiphosphate. In the current study, two enzymes ([2.7.1.148] and [4.6.1.12]) were absent, indicating that other alternative enzymes suitable for these catalytic reactions might exist.

## Discussion

To further understand the destabilization mechanisms of MBF-12, we need to discuss the charge of the functional groups on the bioflocculant as a function of pH. Only organic carboxyl groups and amine groups were especially discussed here, as other groups (e.g., phosphate groups) have much lower concentration on the bioflocculant. The pKa of organic carboxyl groups are 4.1^29^ and the pKa of organic amine groups are 8.8^30^; consequently, we need to examine the charge on the bioflocculant for pH less than pH 4.1, 4.1 to 8.8 and pH greater than pH 8.8.

**Figure 5:**
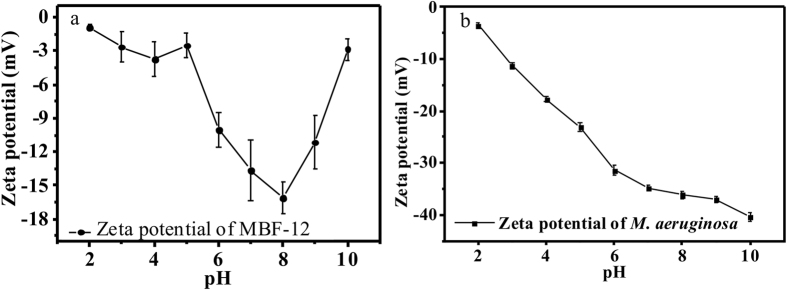
Zeta potential changing of MBF-12 and *M. aeruginosa* versus pH.

The carboxyl groups would be fully protonated at a pH <3.1, and we would expect to see the zeta potential to be positive depending on the surface concentration of amine groups. We do not see a positive zeta potential, but we do see that the zeta potential increased when pH decreasing from 4 to 1 (see Fig. 5). Perhaps there are uncharged oxygen containing functional groups that cause the Zeta potential to be always negative. Nevertheless, the *M. aeruginosa* cells bioflocculation efficiency increased in pH range from 4 to 1, which was consistent with the charge changing trends of the bioflocculant (see Fig. 5).

As the pH increases from 4.1 to 8.8, we do see the zeta potential becomes more negative as the carboxyl group ionize. Consequently, the flocculation mechanism of MBF-12 would be bridging for pH in this range. The amine groups would be protonated for pH values <8.8, and we would expect to see that the flocculation efficiency to decrease with increasing pH from 4.1 to 8.8. However, charge neutralization would not occur because the charge on the algal cells is negative and the charge on the bioflocculant is also negative. Consequently, bridging would be the predominate flocculation mechanism for pH values from 4.1 to 8.8, and the effectiveness of flocculation was observed to decrease as expected as pH increased in this range because of repulsion between the bioflocculant biopolymers and the algae cells.

As the pH increases above 8.8, we see the zeta potential of MBF-12 increased obviously from ~−16 mV to ~−2 mV. A possible explanation is that at high pH values hydrolysis occurs which removes the carboxyl groups from peptidoglycans and lipopolysaccharides under alkaline conditions. And, amine groups, which may lose a proton then resulted bioflocculant become more negatively charged. Furthermore, *M. aeruginosa* cells became more negatively charged as the pH increases, i.e., reached ~−40 mV when pH >8.8 (See Fig. 5). Consequently, the flocculation efficiency decreased with increasing pH due to electrostatic repulsion between the bioflocculant and cells (see Fig. 2). For pH values greater than 8, bridging would still be the predominant flocculation mechanism but the flocculation effectiveness diminished, which was observed, because negative charge on the algal cells increased.

Besides interparticle bridging, biodsorption appears to be another main bioflocculation mechanisms of MBF-12. The basic components of microalgal cell wall are cellulose and glycoproteins^31^, which could strongly be adsorbed by the main components of MBF-12, lipopolysaccharide, peptidoglycan and Isopentenyl-PP (according to the transcriptome analysis). According to Filho^32^, low surface-charge nonionic polymers and the high-molecular-weight should be in the range from 10^5^ to 10^7^ g/mol for effective bridging. The molecular weight of MBF-12, 270 kDa, was just in this range. Furthermore, the Donnan potential on two sides of algal cell walls could adsorb inorganic metal ions for cell metabolisms^33^,^34^,^35^,^36^, which may also facilitate adsorption and interparticle bridging to occur between flocculant MBF-12 and *M. aeruginosa* cells.

Overall, MBF-12 was the biopolymer that with MW as much as 270 kDa. It should be originated from the extracellular substances of *Citrobacter sp. AzoR-1*, according to our extraction method as given in Supporting Information (SI-2). MBF-12 is comprised of high-molecular-weight polymers, with both of positive groups and negative groups, and they can flocculate *M. aeruginosa* cells mainly through interparticle bridging process and biosorption as shown in Figure S2 33.

The vigorous bioflocculant synthesis in *Citrobacter* sp*. AzoR-1*, especially the prevalence of mature lipopolysaccharide synthase transcripts which has never been reported before in *Citrobacteria* transcriptomes, was considered to be resulted by feeding the bacteria with the microalgal biomass substrate. The transcriptome analysis of the microalgal substrate culture revealed that the genes related to the MBF-12 synthesis of *Citrobacter* sp*. AzoR-1* were positively regulated. As expected, the *Citrobacter* sp*. AzoR-1* transcriptome contained many expressed sequence tags (ESTs) associated with the polysaccharide biosynthesis system, which was consistent with previous research results^4^,^5^,^37^. Interestingly, the *Citrobacter* sp*. AzoR-1* transcriptome data embraced many genes associated with terpenoid backbone metabolism.

In this work, the efficiency of MBF-12 for *M. aeruginosa* harvesting could reach ~95% under the optimized condition. Even though the commercial inorganic flocculants, such as Al_2_(SO_4_)_3_, FeCl_3_ and Polyaluminum chloride (PAC), showed similar harvesting efficiencies for *M. aeruginosa* (see Figure S4), however, they may cause micralgal cell rupture and drain, and thus increase algal toxins in the aquatic environment^38^,^39^. These commercial flocculants are usually artificial synthesized, in contrast, MBF-12 was produced by reutilizing the harvested microalgal biomass via a bioflocculant-producing bacteria cultivation. This implied MBF-12 was potentially more economical. Furthermore, MBF-12 was proved to be ion independent during flocculation (as confirmed in Fig. 1), whereas the previous reported bioflocculants were normally cation-dependent^40^,^41^. As a result, the metal cation could be saved when using MBF-12 for microalgae flocculation. Guo *et al*.^42^ and Alam *et al*.^43^ also reported the high efficiency of extracellular biopolymers on microalgae harvesting (90% in flocculation efficiency), but they did not reutilize the harvested biomass in their work^44^,^45^.

Bioresources inner-recycling, between bioflocculation of *Microcystis aeruginosa* and its reutilization as a substrate for bioflocculant production, was achieved in this work. By this means, the bioflocculation of *Microcystis aeruginosa* reached as much as 95%, with the bioflocculant produced from *Citrobacter sp. AzoR-1* by the harvested *Microcystis aeruginosa* biomass.

## Methods

The bioflocculant-producing strain was isolated from an activated sludge of a secondary wastewater treatment plant in Changchun, China. The 16 S rDNA sequence of the strain was 99.9% similar to *Citrobacter* sp*. AzoR-1*, as identified by Sangon Biotechnology Co., Ltd (Shanghai, China). The sequencing result is given in Supplementary Information SI-1.

For comparison, the composition of the previously reported bioflocculant production medium^14^ (hereafter called PT-1) was used (g/L) for *Citrobacter* sp*. AzoR-1* cultivation, 20 glucose, 0.5 yeast extract, 0.5 urea, 5 K_2_HPO_4_, 2 KH_2_PO_4_, 0.2 MgSO_4_ and 0.1 NaCl. The initial pH of the medium was adjusted to 7.0.

When using microalgal biomass as substrates for bioflocculant production, we optimized the medium components by supplementing extra carbon and nitrogen to 10 g/L *M. aeruginosa* biomass (see details in Table 1). The biomass concentration refers to the wet weight concentration after centrifugation and before high-pressure steam sterilization. After sterilization and inoculation of the media, the strains were cultured in a rotary shaker at 30 °C and 150 rpm for 72 h.

The methods used to prepare the bioflocculant are given in Supplementary Information SI-2. The microbial flocculant of *Citrobacter* sp*. AzoR-1* was termed MBF-12.

*M. aeruginosa* was cultured for 10 days in BG11 medium to a concentration of ~10^9^ cells per liter for the flocculation study. *M. aeruginosa* cells were washed twice with distilled water prior to the flocculation experiments to remove the influence of ions from the various culture media. The initial *M. aeruginosa* concentration was determined from the optical density at 680 nm (OD_680_) using a UV-vis spectrophotometer (Model-T6, PGENERAL Ltd., Beijing, China).

Beakers containing the mixture of *M. aeruginosa* solution and MBF-12 were shaken in an orbital shaker (Model-HZQ-X100, HDL APPARATUS Ltd., Hangzhou, China). After the addition of the bioflocculant, the biomass was stirred at high *G* values of ~350 s^−1^ for rapid mixing for 1 minute, and then low *G* value of ~19 s^−1^ for 5 minutes to promote the mixing bioflocculation of *M. aeruginosa* cells. Finally, the mixture was settled from 0 to 1.71 hours. Culture broth without bioflocculant was utilized as a control.

The removal efficiency of *M. aeruginosa* was determined using the following calculation, *removal efficiency (%*) = (*A* − *B*)/*A* × *100*, where A is the OD_680_ value of supernatant of the sample and B is the OD_680_ value of supernatant of the control. The optical density of the supernatant was determined at the half height of the clarified culture.

A central composite design (CCD) and response surface methodology (RSM) were applied to optimize the MBF-12 flocculation conditions in this study (see details in Supplementary Informtion SI-3). Optimization of the operating conditions was conducted using the quadratic models of the experimental design. The removal efficiency of *M. aeruginosa* cells was determined by testing five optimum solutions provided by the models. The solutions were labeled from 1–5 and were selected and verified under selected optimum conditions (see details in Table S4).

The Zeta potential of MBF-12 was determined using a Malvern nano ZS90 analyzer (Malvern Instruments Ltd, Worcestershire, UK). A Fourier-transform infrared spectrometer (Nicolet 6700, Thermo Fisher Scientific, Waltham, USA) was used for MBF-12 detection after vacuum drying pretreatment. Fluorescence excitation emission matrix (EEM) analyses were conducted with a fluorescence spectrophotometer (Hitachi F-7000, Hitachi Inc., Tokyo, Japan) at a scan rate of 240 nm/min and an excitation/emission slit bandwidth of 2.5 nm. The scanning field was set at excitation from 220 to 400 nm and emission spectra from 300 to 550 nm.

Total RNA was used for the complementary DNA (cDNA) library construction and was extracted using the RNeasy MinElute Cleanup Kit (Qiagen Inc., Hilden, Germany) according to the manufacturer’s instructions^46^. The *Citrobacter* sp*. AzoR-1* was cultured axenically in Culture-C; cells were harvested five days after inoculation, and the samples were immediately frozen in liquid nitrogen. Then, the samples were ready for transcription analyses to reveal the sequences underlying the bioflocculant production (see detailed procedures in Supplementary Information SI-4).

## Additional Information

**How to cite this article:** Xu, L. *et al*. Bioresources inner-recycling between bioflocculation of *Microcystis aeruginosa* and its reutilization as a substrate for bioflocculant production. *Sci. Rep.* **7**, 43784; doi: 10.1038/srep43784 (2017).

**Publisher's note:** Springer Nature remains neutral with regard to jurisdictional claims in published maps and institutional affiliations.

## Supplementary Material

Supplementary Information

## Figures and Tables

**Table 1:** Supplemented components when using microalgal biomass to produce bioflocculant.

Culture No.	Supplemented components (g/L)
Culture-A	*/*(using the biomass as the entire culture)
Culture-B	glucose, 20 (with extra carbon than A)
Culture-C	glucose, 10 (with less of the extra carbon than B)
Culture-D	glucose, 10; yeast extract, 0.5 (with extra organic nitrogen)
Culture-E	glucose, 10; yeast extract, 0.5; urea, 0.5 (with extra inorganic nitrogen)
Culture-F	urea, 0.5 (with only extra inorganic nitrogen)

*All cultures included 10 g/L *M. aeruginosa* wet biomass.
