# Supplementary material for: Bioresources inner-recycling between bioflocculation of Microcystis aeruginosa and its reutilization as a substrate for bioflocculant production
Source: Sci Rep. 2017 Mar 2;7:43784. doi: 10.1038/srep43784 (PMC5333146; doi:10.1038/srep43784)
Supplement: Supplementary Information [file srep43784-s1.doc]

**Bioresources inner-recycling between bioflocculation of *Microcystis aeruginosa* and its reutilization as a substrate for bioflocculant production**

*Liang Xu^1,2^, Mingxin Huo^1^, Caiyun Sun^1^, Xiaochun Cui^1^, Dandan Zhou*^,1^, John C. Crittenden^1^,*^3^*, Wu Yang^*,1^*

^1^ School of Environment, Northeast Normal University, Changchun 130117, China

^2^ Jilin Institute of Chemical Technology, Jilin, 132022, China

^3^ Brook Byers Institute for Sustainable Systems, and School of Civil & Environmental Engineering, Georgia Institute of Technology, Atlanta, GA 30332, United States.

**Corresponding author**

*(D. Zhou) Phone: +86 13604331805; fax: +86 43189165610

E-mail: [zhoudandan415@163.com](mailto:zhoudandan415@163.com)

*(W. Yang) Phone: +86 13756478864; fax: +86 43189165610

E-mail: yangw104@nenu.edu.cn

**Supplementary information**

22 Pages including cover page

5 Tables

4 Figures

**SI-1 sequencing results of *Citrobacter* sp*. AzoR-1***

PCR amplification of 16S rDNA was identified by Sangon Biotechnology (Shanghai, China Co., Ltd) and the sequencing results of are as follows,

GCAGTCGAACGGTAGCACAGAGGAGCTTGCTCCTTGGGTGACGAGTGGCGGACGGGTGAGTAATGTCTGGGAAACTGCCCGATGGAGGGGGATAACTACTGGAAACGGTAGCTAATACCGCATAACGTCGCAAGACCAAAGAGGGGGACCTTCGGGCCTCTTGCCATCGGATGTGCCCAGATGGGATTAGCTAGTAGGTGGGGTAACGGCTCACCTAGGCGACGATCCCTAGCTGGTCTGAGAGGATGACCAGCCACACTGGAACTGAGACACGGTCCAGACTCCTACGGGAGGCAGCAGTGGGGAATATTGCACAATGGGCGCAAGCCTGATGCAGCCATGCCGCGTGTATGAAGAAGGCCTTCGGGTTGTAAAGTACTTTCAGCGAGGAGGAAGGTGTTGTGGTTAATAACCGCAGCAATTGACGTTACTCGCAGAAGAAGCACCGGCTAACTCCGTGCCAGCAGCCGCGGTAATACGGAGGGTGCAAGCGTTAATCGGAATTACTGGGCGTAAAGCGCACGCAGGCGGTCTGTCAAGTCGGATGTGAAATCCCCGGGCTCAACCTGGGAACTGCATCCGAAACTGGCAGGCTAGAGTCTTGTAGAGGGGGGTAGAATTCCAGGTGTAGCGGTGAAATGCGTAGAGATCTGGAGGAATACCGGTGGCGAAGGCGGCCCCCTGGACAAAGACTGACGCTCAGGTGCGAAAGCGTGGGGAGCAAACAGGATTAGATACCCTGGTAGTCCACGCCGTAAACGATGTCGACTTGGAGGTTGTGCCCTTGAGGCGTGGCTTCCGGAGCTAACGCGTTAAGTCGACCGCCTGGGGAGTACGGCCGCAAGGTTAAAACTCAAATGAATTGACGGGGGCCCGCACAAGCGGTGGAGCATGTGGTTTAATTCGATGCAACGCGAAGAACCTTACCTACTCTTGACATCCAGAGAACTTAGCAGAGATGCTTTGGTGCCTTCGGGAACTCTGAGACAGGTGCTGCATGGCTGTCGTCAGCTCGTGTTGTGAAATGTTGGGTTAAGTCCCGCAACGAGCGCAACCCTTATCCTTTGTTGCCAGCGATTCGGTCGGGAACTCAAAGGAGACTGCCAGTGATAAACTGGAGGAAGGTGGGGATGACGTCAAGTCATCATGGCCCTTACGAGTAGGGCTACACACGTGCTACAATGGCATATACAAAGAGAAGCGACCTCGCGAGAGCAAGCGGACCTCATAAAGTATGTCGTAGTCCGGATTGGAGTCTGCAACTCGACTCCATGAAGTCGGAATCGCTAGTAATCGTGGATCAGAATGCCACGGTGAATACGTTCCCGGGCCTTGTACACACCGCCCGTCACACCATGGGAGTGGGTTGCAAAAGAAGTAGGTAGCTTAACCTTCGGGAGGGCGCTTACCACTTTGTGATTCATGAC


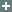
        domain Bacteria  (0/20/1255860)

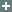
            phylum "Proteobacteria"  (0/20/349465)

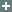
                class Gammaproteobacteria  (0/20/158841)

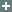
                    order "Enterobacteriales"  (0/20/35391)

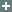
                        family Enterobacteriaceae  (0/20/35391)

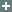
                            genus Citrobacter  (0/20/1445)
[S000653211](http://rdp.cme.msu.edu/seqmatch/seqmatch_seqrecorddetail.jsp?seqid=S000653211)      0.999  Citrobacter sp. AzoR-1; DQ279748

**SI-2 Preparation of bioflocculant (MBF-12)**

*Citrobacter sp. AzoR-1* was inoculated into a 150 mL flask containing 50 mL PT-1 medium. The bioflocculant produced by *Citrobacter sp. AzoR-1*, termed MBF-12, was produced by shaking the flask under 30 °C at 150 rpm for 72 h. Cell-free supernatant was obtained by centrifugation at 7000 rpm for 30 min. Then, two volumes of cold ethanol (at 4 °C) were added to 1 L culture broth for bioflocculant extraction. Following centrifugation at 8000 rpm for 30 min, the precipitate was collected and dissolved in 100 mL of 0.5 M NaCl solution after 3 h. Finally, two volumes of cold ethanol were then added to obtain the precipitate. The precipitate was washed with ethanol, dissolved in 5 mL of deionized water and vacuum-dried resulting MBF-12 obtained in 1L culture broth.

**SI-3 Optimization of *M. aeruginosa* flocculation with Response Surface Quadratic Model**

Evaluations were conducted to assess the effects of different flocculation conditions (temperature, pH, settling time, MBF-12 dosage) on the flocculation efficiency. The initial pH was adjusted to 3.0-11.0 (± 0.2) using 0.1 M HCl or NaOH.

Design Expert Software (version 8.0) was employed for the statistical design of the experiments and data analysis. A central composite design (CCD) and response surface methodology (RSM) were applied to optimize the four factors in this study: MBF-12 dosage, temperature, settling time and pH. Independent variables for the flocculation conditions and their experimental levels are given in Table S1.

The response variable (y) that represented the removal efficiency was derived from a second-order model and was calculated using the following quadratic polynomial equation,

*y (removal efficiency) = + 85.02 - 0.15 × A - 22.60 × B + 10.23 × C + 20.04 × D + 1.78 × A× B - 0.43 × A× C - 1.30 × A × D - 0.019 × B × C - 7.61 × B × D + 0.22 × C × D + 3.55 × A^2^ - 19.82 × B^2^ - 12.56 × C^2^ - 17.57 × D^2^*,

where the removal efficiency is the predicted response and the independent factors A, B, C and D are the temperature, pH, settling time (G value was 0 s^-1^) and MBF-12 dosage, respectively.

Narrower ranges for the MBF-12 dosage, temperature, settling time (the G value was 0 s^-1^) and pH were determined prior to designing the experimental runs. These ranges were set at 2.93-17.70 mg/L, 0.29-1.71 hours, 5-35 °C and 2-12 for flocculation of 10^9^ cells per liter (OD_680_ = 3.0±0.5), respectively. The biomass was harvested by decanting the water after flocculation. After the addition of the bioflocculant, the biomass was stirred at high G values of ~350 s^-1^ for rapid mixing of the flocculant MBF-12 for 1 minutes, and then low G value of ~19 s^-1^ for 5 minutes to promote the mixing flocculation of *M. aeruginosa* cells in a vortex oscillator. The settling times tested ranged from 0 to 1.7 hours. The settling time (G value was 0 s^-1^) refers to the time taken for the biomass to settle to the bottom of the beaker after the flocculation step. The removal efficiencies at different cultivation conditions are shown in Table S2.

Experimental data were analyzed by ANOVA with all factors following second-order effects, yielding a quadratic model for flocculation efficiency. The model may adequately describe the response based on the p-value < 0.0001, far less than 0.05. The insignificant terms (p-value > 0.05) are removed from final expression of the model ^a^. LOGIT transformation was utilized with upper limit as 100 to bind the response for Y (flocculation efficiency) to 100%. The model, in forms of the coded factors of the flocculation process. As shown in Table S2 (Supplementary), Adequate precision evaluates the ratios of signal and noise ratio with a ratio greater than 4 as desirable. The model’s ability to precisely describe the process was confirmed as the ratio was 13.24 (Table S3) and the lack of fit value was insignificant relative to pure error as the p-value was less than 0.05. Value of correlation coefficient (R^2^) obtained was 0.88, close to R^2^ adj. (0.94), indicating alignment between the predicted and observed values of the experiments.

**SI-4 Transcription analyses procedure**

Total RNA was used for the complementary DNA (cDNA) library construction and was extracted using the RNeasy MinElute Cleanup Kit (Qiagen Inc., Hilden, Germany) according to the manufacturer’s instructions. The *Citrobacter* sp*. AzoR-1* was cultured axenically in Culture-C; cells were harvested five days after inoculation, and the samples were immediately frozen in liquid nitrogen. Contaminating DNA was eliminated using RNase-Free DNase I (Takara Inc., Kusatsu, Japan), and the rRNA was eliminated from the total DNase I using RNA with the Ribo-Zero™ Magnetic Kit (Epicentre Inc., Wisconsin, USA).

We took 100 ng of rRNA-depleted RNA and applied it to an NEB Next ® Ultra^TM^ Directional RNA Library Prep Kit for Illumina (NEB Ltd, Massachusetts, USA) to build a library. After building the library, we ensured its quality via high-sensitivity DNA chip detection. We then performed cluster generation using 10 ng of the library, the TruSeq PE Cluster Kit (Illumina Inc, California, USA) and the cBot system. The library DNA was then sequenced using an Illumina HiSeq^TM^2500 system (Illumina Inc, California, USA). The clean transcriptome reads were analyzed without a reference genome. Non-redundant consensus sequences were generated by assembling the clean reads using the Trinity software program, and the assembled sequences were employed as reference sequences for the subsequent pathway analysis.

High-throughput sequencing of transcriptome of *Citrobacter Aroz-1* from Culture-C generated 7,328,816 cDNA reads. The majority of the cDNA reads were 200–300 bases in length, and the average read length was 200 bases. By assembling the cDNA reads, 4,961 non-redundant sequences were retrieved; the average length of these sequences was 296 bases. The majority of the non-redundant sequences consisted of a single cDNA read, while some non-redundant sequences consisted of more than 4,961 cDNA reads. The average GC content using all non-redundant sequences was 48.8%. For the 4,961 non-redundant sequences, the gene functions were successfully predicted based on their homology with previously characterized genes from other organisms.

**Table S1. Independent variables for the flocculation conditions and their experimental levels**

| Independent variables | Symbols | Code levels | | | | |
| --- | --- | --- | --- | --- | --- | --- |
|  |  | -1 | -2 | 0 | 1 | 2 |
| Temperature (˚C) | A | 5.86 | 10 | 20 | 30 | 34.14 |
| pH | B | 2.05 | 3.5 | 7 | 10.5 | 11.95 |
| Settling time (hour) | C | 0.29 | 0.5 | 1 | 1.5 | 1.71 |
| MBF-12 dosage (mg/L) | D | 2.93 | 5 | 10 | 15 | 17.07 |

**Table S2.** Experimental design and results of the central composite design

| Run | Variables | | | | Removal efficiency (%) |
| --- | --- | --- | --- | --- | --- |
|  | Temperature (˚C) | pH | Flocculation time (hour) | MBF-12 dosage (mg) |  |
|  |  |  |  |  |  |
| 1 | 30 | 3.5 | 0.5 | 15 | 77.67 |
| 2 | 20 | 7 | 1 | 17.07 | 95.37 |
| 3 | 20 | 7 | 1 | 10 | 85.12 |
| 4 | 20 | 7 | 0.29 | 10 | 25.93 |
| 5 | 20 | 7 | 1 | 10 | 85.54 |
| 6 | 30 | 3.5 | 1.5 | 5 | 44.35 |
| 7 | 10 | 10.5 | 0.5 | 15 | 16.21 |
| 8 | 30 | 3.5 | 0.5 | 5 | 24.81 |
| 9 | 10 | 3.5 | 1.5 | 15 | 91.7 |
| 10 | 30 | 10.5 | 0.5 | 15 | 15.39 |
| 11 | 20 | 2.05 | 1 | 10 | 85.91 |
| 12 | 10 | 3.5 | 0.5 | 15 | 85.22 |
| 13 | 5.86 | 7 | 1 | 10 | 89.97 |
| 14 | 20 | 7 | 1 | 10 | 84.96 |
| 15 | 34.14 | 7 | 1 | 10 | 94.14 |
| 16 | 10 | 3.5 | 1.5 | 5 | 47.34 |
| 17 | 20 | 7 | 1 | 2.93 | 4.28 |
| 18 | 20 | 7 | 1 | 10 | 85.25 |
| 19 | 10 | 10.5 | 0.5 | 5 | 2.53 |
| 20 | 10 | 3.5 | 0.5 | 5 | 23.89 |
| 21 | 20 | 7 | 1.71 | 10 | 93.75 |
| 22 | 30 | 10.5 | 0.5 | 5 | 9 |
| 23 | 30 | 10.5 | 1.5 | 15 | 39.38 |
| 24 | 20 | 11.95 | 1 | 10 | 4.75 |
| 25 | 30 | 3.5 | 1.5 | 15 | 82.69 |
| 26 | 30 | 10.5 | 1.5 | 5 | 11.36 |
| 27 | 20 | 7 | 1 | 10 | 84.99 |
| 28 | 10 | 10.5 | 1.5 | 5 | 9.76 |
| 29 | 20 | 7 | 1 | 10 | 84.45 |
| 30 | 10 | 10.5 | 1.5 | 15 | 36.82 |

**Table S3.** ANOVA analysis for Response Surface Quadratic Model

| Source | Sum of squares | DF | Mean square | F value | P value |
| --- | --- | --- | --- | --- | --- |
| Model | 34064.49 | 14 | 2433.18 | 16.78 | < 0.0001 |
| B-pH | 10215.37 | 1 | 10215.37 | 70.44 | < 0.0001 |
| C-Flo time | 2092.79 | 1 | 2092.79 | 14.43 | 0.0017 |
| D-Flo dosage | 8034.52 | 1 | 8034.52 | 55.41 | < 0.0001 |
| BD | 926.39 | 1 | 926.39 | 6.39 | 0.0232 |
| B^2 | 3665 | 1 | 3665 | 25.27 | 0.0002 |
| C^2 | 1472.49 | 1 | 1472.49 | 10.15 | 0.0061 |
| D^2 | 2880.31 | 1 | 2880.31 | 19.86 | 0.0005 |
| Residual | 2175.18 | 15 | 145.01 |  |  |
| Lack of Fit | 2174.53 | 10 | 217.45 | 1667.37 | < 0.0001 |
| Pure Error | 0.65 | 5 | 0.13 |  |  |
| R-Squared | 0.94 |  |  |  |  |
| Adj R-Squared | 0.88 |  |  |  |  |
| Adeq Precision | 13.24 |  |  |  |  |

**Table S4.** Verification experiments at optimum conditions

| Test no. | Variables | | | | Removal efficiency (%) | | | | |
| --- | --- | --- | --- | --- | --- | --- | --- | --- | --- |
|  | Temperature (˚C) | pH | Settling time (hour) | MBF-12 dosage (mg/L) | Predicted values | Observed values | | Standard deviation | |
| 1 | 20 | 7 | 1.18 | 12.84 | 92.82 | | 93.87 | | ±0.74 |
| 2 | 15 | 7 | 1.2 | 13 | 94.28 | | 91.68 | | ±1.84 |
| 3 | 30 | 7 | 1.2 | 13 | 95.3 | | 94.48 | | ±0.58 |
| 4 | 25 | 5 | 1.27 | 11.76 | 99.73 | | 97.21 | | ±1.78 |
| 5 | 15 | 6 | 1 | 12.7 | 98.29 | | 96.69 | | ±1.13 |

| **Table S5.** Enzymes potentially associated with biosynthesis of the polysaccharide-derived bioflocculant. | | | | | | | | |
| --- | --- | --- | --- | --- | --- | --- | --- | --- |
| **gene name** | | **EC number** | **Enzym name** | **lenths** | | **reads** | **rpkm** | |
| **Polyketide sugar unit biosynthesis** | | | | | | | | |
| rfbD, rmlD | [1.1.1.133] | | dTDP-4-dehydrorhamnose reductase | | 495 | 1404 | 37.98 | |
| **Lipopolysaccharide biosynthesis** | | | | | | | | |
| waaL, rfaL | [2.4.1.-] | | hypothetical protein | | 90 | 49 | 10.54 | |
| waaP, rfaP | [2.7.1.-] | | lipopolysaccharide core heptose(I) kinase rfaP | | 87 | 41 | 8.31 | |
| waaG, rfaG | [2.4.1.-] | | glucosyltransferase I RfaG | | 813 | 65 | 13.22 | |
| waaC, rfaC | [2.4.-.-] | | lipopolysaccharide heptosyltransferase 1 | | 249 | 238 | 33.98 | |
| gmhC, hldE, waaE, rfaE | [2.7.1.167 2.7.7.70] | | bifunctional heptose 7-phosphate kinase/heptose 1-phosphate adenyltransferase | | 1002 | 41 | 8.08 | |
| gmhA, lpcA | [5.3.1.28] | | phosphoheptose isomerase | | 687 | 132 | 36.75 | |
| gmhD, rfaD | [5.1.3.20] | | ADP-L-glycero-D-mannoheptose-6-epimerase | | 399 | 383 | 43.46 | |
| gmhB | [3.1.3.82 3.1.3.83] | | D,D-heptose 1,7-bisphosphate phosphatase | | 165 | 182 | 42.60 | |
| kdsA | [2.5.1.55] | | 2-dehydro-3-deoxyphosphooctonate aldolase | | 363 | 328 | 36.47 | |
| lpxB | [2.4.1.182] | | lipid-A-disaccharide synthase | | 1020 | 105 | 12.94 | |
| htrB | [2.3.1.-] | | lipid A biosynthesis lauroyl acyltransferase | | 831 | 32 | 8.60 | |
| msbB | [2.3.1.-] | | lipid A biosynthesis (KDO)2-(lauroyl)-lipid IVA acyltransferase | | 663 | 28 | 8.65 | |
| kdtA, waaA | [2.4.99.12 2.4.99.13] | | 3-deoxy-D-manno-octulosonic-acid transferase | | 189 | 5 | 4.81 | |
| waaO, rfaI | [2.4.1.-] | | hypothetical protein | | 204 | 5 | 4.90 | |
| lpxK | [2.7.1.130] | | tetraacyldisaccharide 4'-kinase | | 204 | 5 | 4.62 | |
| **Peptidoglycan biosynthesis** | | | | | | | | |
| pbp2B, penA | [2.3.2.-] | | hypothetical protein | | 267 | 5 | 3.56 | |
| mrdA |  | | penicillin-binding protein 2 | | 315 | 46 | 11.77 | |
| murB | [1.3.1.98] | | UDP-N-acetylenolpyruvoylglucosamine reductase | | 573 | 137 | 25.98 | |
| dacC, dacA, dacD | [3.4.16.4] | | D-alanyl-D-alanine carboxypeptidase | | 465 | 306 | 46.13 | |
| pbpC | [2.4.1.-] | | penicillin-binding protein 1C | | 693 | 183 | 30.37 | |
| ftsI |  | | peptidoglycan synthase ftsI | | 951 | 838 | 157.64 | |
| ddl | [6.3.2.4] | | D-alanine--D-alanine ligase | | 681 | 100 | 19.55 | |
| murE | [6.3.2.13] | | UDP-N-acetylmuramoylalanyl-D-glutamate--2, 6-diaminopimelate ligase | | 909 | 7 | 5.11 | |
| ddl | [6.3.2.4] | | D-alanine--D-alanine ligase | | 333 | 39 | 8.40 | |
| bacA | [3.6.1.27] | | undecaprenyl pyrophosphate phosphatase | | 198 | 41 | 10.38 | |
| murG | [2.4.1.227] | | undecaprenyldiphospho-muramoylpentapeptide beta-N-acetylglucosaminyltransferase | | 228 | 9 | 7.70 | |
| mrcA | [2.4.1.- 3.4.-.-] | | penicillin-binding protein 1a | | 213 | 20 | 5.40 | |
| bacA | [3.6.1.27] | | undecaprenyl pyrophosphate phosphatase | | 333 | 25 | 10.94 | |
| murD | [6.3.2.9] | | UDP-N-acetylmuramoyl-L-alanyl-D-glutamate synthetase | | 192 | 5 | 4.56 | |
| mraY | [2.7.8.13] | | phospho-N-acetylmuramoyl-pentapeptide-transferase | | 201 | 5 | 5.00 | |
| vanY | [3.4.16.4] | | peptidase M15 | | 201 | 7 | 5.49 | |
| mrcB | [2.4.1.129 3.4.-.-] | | bifunctional glycosyl transferase/transpeptidase | | 1260 | 198 | 28.30 | |
| mrcB | [2.4.1.129 3.4.-.-] | | bifunctional glycosyl transferase/transpeptidase | | 516 | 67 | 10.91 | |
| murA | [2.5.1.7] | | UDP-N-acetylglucosamine 1-carboxyvinyltransferase | | 675 | 161 | 16.15 | |
| ddl | [6.3.2.4] | | D-alanyl-alanine synthetase A | | 162 | 46 | 14.62 | |
| murC | [6.3.2.8] | | UDP-N-acetylmuramate-L-alanine ligase | | 420 | 15 | 9.73 | |
| dacC, dacA, dacD | [3.4.16.4] | | D-alanyl-D-alanine carboxypeptidase | | 276 | 8 | 7.56 | |
| dacB |  | | D-alanyl-D-alanine carboxypeptidase/endopeptidase | | 201 | 20 | 7.16 | |
| mrcA | [2.4.1.- 3.4.-.-] | | penicillin-binding protein 1A/1B | | 180 | 9 | 6.57 | |
| mraY | [2.7.8.13] | | phospho-N-acetylmuramoyl-pentapeptide-transferase | | 366 | 8 | 5.88 | |
| murJ, mviN |  | | membrane protein | | 207 | 6 | 5.49 | |
| murF | [6.3.2.10] | | UDP-N-acetylmuramoyl-tripeptide--D-alanyl-D-alanine ligase | | 120 | 8 | 4.77 | |
| **Terpenoid backbone biosynthesis** | | | | | | | |  |
| dxs | [2.2.1.7] | | 1-deoxy-D-xylulose-5-phosphate synthase | | 291 | 4 | 3.13 |  |
| ispB | [2.5.1.90] | | octaprenyl-diphosphate synthase | | 324 | 35 | 13.79 |  |
| idsA | [2.5.1.1 2.5.1.10 2.5.1.29] | | octaprenyl-diphosphate synthase | | 2145 | 15 | 9.64 |  |
| idi, IDI | [5.3.3.2] | | isopentenyl-diphosphate Delta-isomerase | | 711 | 104 | 29.83 |  |
| dxr | [1.1.1.267] | | 1-deoxy-D-xylulose 5-phosphate reductoisomerase | | 315 | 513 | 31.86 |  |
| gcpE, ispG | [1.17.7.1 1.17.7.3] | | 4-hydroxy-3-methylbut-2-en-1-yl diphosphate synthase | | 471 | 67 | 14.98 |  |
| ispH, lytB | [1.17.1.2] | | 4-hydroxy-3-methylbut-2-enyl diphosphate reductase | | 381 | 46 | 11.56 |  |
| ispD | [2.7.7.60] | | 2-C-methyl-D-erythritol 4-phosphate cytidylyltransferase | | 414 | 17 | 8.20 |  |
| dxs | [2.2.1.7] | | 1-deoxy-D-xylulose 5-phosphate synthase | | 207 | 11 | 7.13 |  |
| ispA | [2.5.1.1 2.5.1.10] | | geranyltranstransferase | | 324 | 11 | 7.87 |  |
| ispE | [2.7.1.148] | | 4-diphosphocytidyl-2C-methyl-D-erythritol kinase | | 240 | 5 | 4.86 |  |




**Figure S1.** Effect of four single variables on removal efficiency, which including: A temperature, B pH, C settling time (G value was 0 s^-1^) and D MBF-12 dosage. ● Verified removal efficiency under selected conditions.


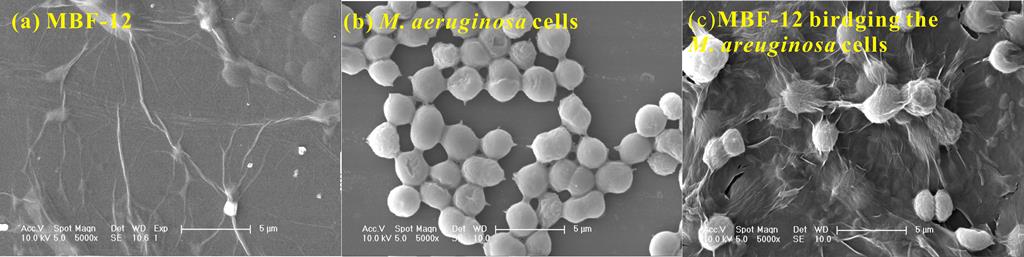


**Figure S2.** (a) Scanning electron microscope of MBF-12 form Culture-C. (b) Scanning electron microscope of *M. aeruginosa* cells. (c) Scanning electron microscope of MBF-12 bridging the *M. aeruginosa* cells.




**Figure S3.** Growth and bioflocculant productivity vs. cultivation time. (a) Bioflocculant productivity in PT-1 Culture; (b) Bioflocculant productivity in Culture-C. Dry *Citrobacter Aroz-1* cell dry weight; Bioflocculant productivity (dry weight).


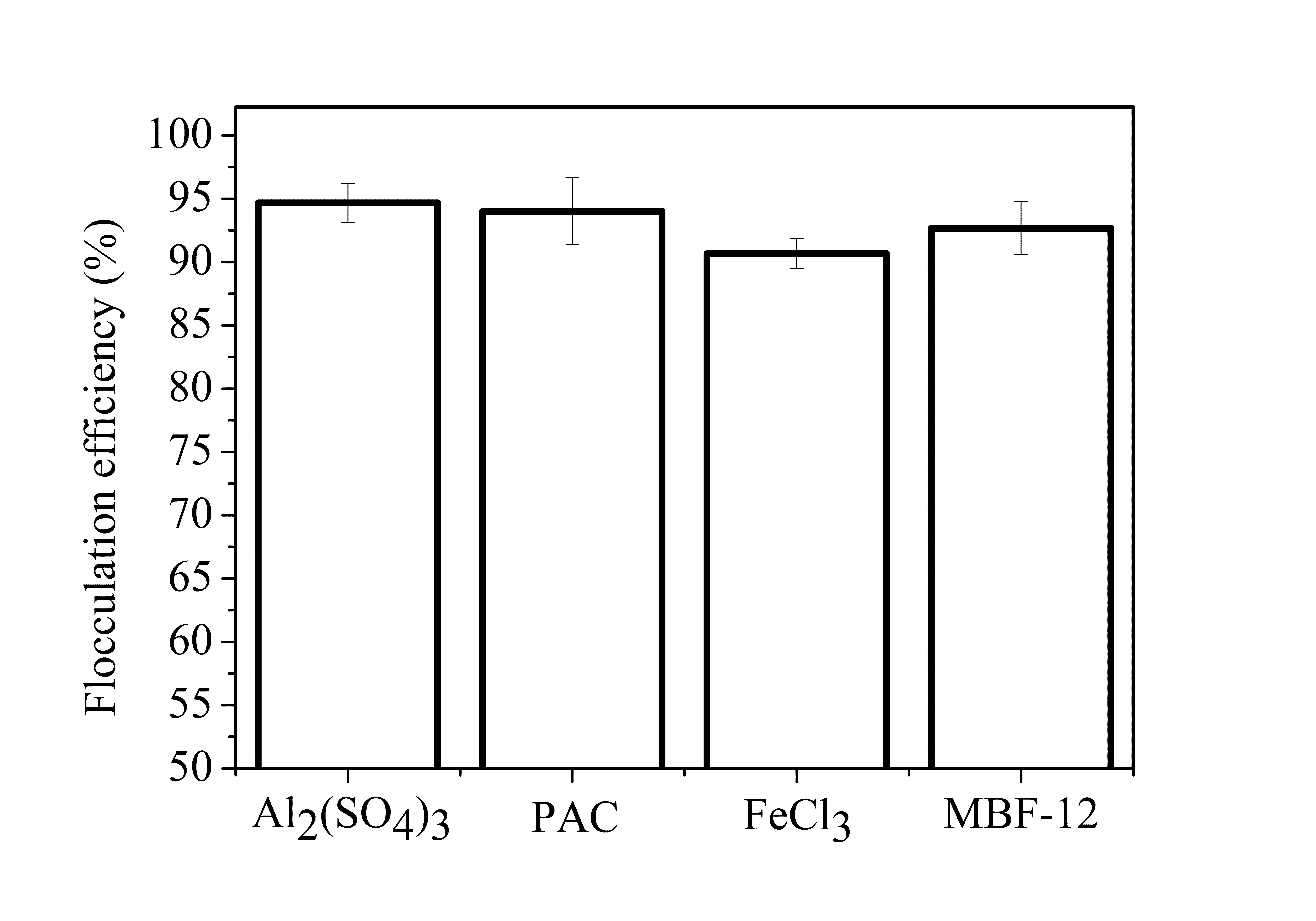


**Figure S4.** Flocculation efficiency of MBF-12 on *M. aeruginosa* in comparison with the popular commercial flocculants of Al_2_(SO_4_)_3_, FeCl_3_ and PAC. The flocculant dosages are all 0.1 g L^-1^, and the flocculation conditions are the same with that given in Methods.

- **References**

(a) Mirmohseni, A., Seyed Dorraji, M. S., Figoli, A., Tasselli, F. Chitosan hollow fibers as effective biosorbent toward dye: Preparation and modeling*. Bioresource Technolo*gy 121, 212-220 (2012).
